# Chiral gold nanoparticles manipulate osteoimmune microenvironment via macrophage autophagy for bone regeneration

**DOI:** 10.1016/j.mtbio.2025.102131

**Published:** 2025-07-25

**Authors:** Jiaolong Wang, Ning Gao, Junchao Wei, Lan Liao

**Affiliations:** a School of Stomatology, Jiangxi Medical College, Nanchang University, Nanchang, 330006, China; b Jiangxi Province Key Laboratory of Oral Diseases & Jiangxi Province Clinical Research Center for Oral Diseases, Nanchang, 330006, China; c School of Chemistry and Chemical Engineering, Nanchang University, Nanchang, 330031, China; d The First Affiliated Hospital, Jiangxi Medical College, Nanchang University, Nanchang, 330006, China

**Keywords:** Chirality, Gold nanoparticles, Autophagy, Macrophage, Bone regeneration

## Abstract

The immune microenvironment orchestrates bone regeneration, which can be manipulated by macrophage autophagy. Herein, gold nanoparticles with L/D (left-handed or right-handed)-chirality (L/D-AuNPs) were synthesized with chiral glutathione ligands to stereoselectively regulate macrophage autophagy, and further manipulate immune microenvironment for bone regeneration. Notably, L-AuNPs exhibited superior macrophage uptake efficiency and higher autophagy level compared to D-AuNPs. Meanwhile, L-AuNPs improved the osteogenic microenvironment, further promoting bone regeneration. In addition, the *in vivo* results showed that the healing of skull defect was significantly enhanced by L-AuNPs. These findings demonstrated that chiral AuNPs can stereoselectively regulate macrophage autophagy and open an avenue for applications of chiral nanoparticles in osteoimmunity.

## Graphical abstract

**Figure:**
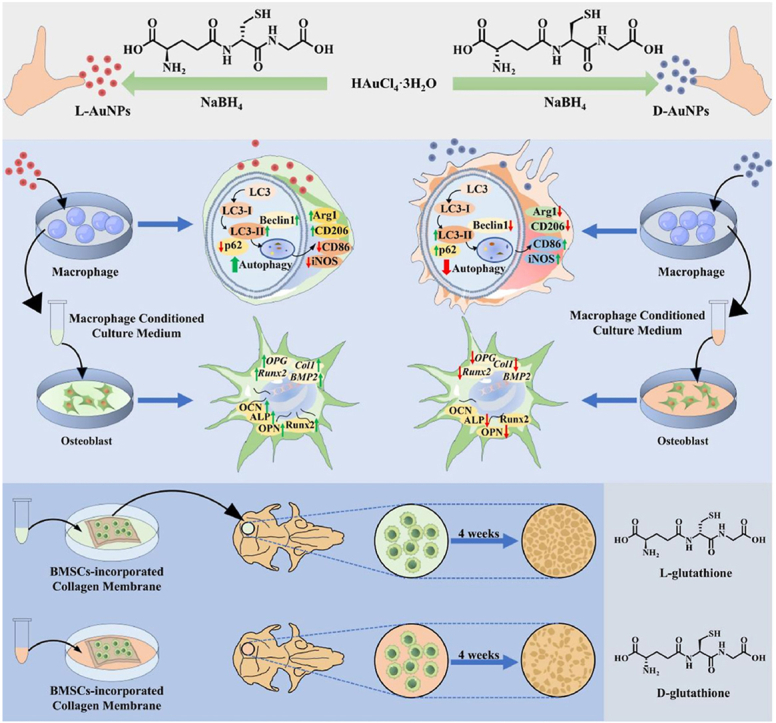

## 1 Introduction

The immune microenvironment orchestrates bone regeneration through inflammation modulation, cell recruitment, and tissue remodeling during bone defects repair [[1], [2], [3]]. Among various immune cells, macrophages are key mediator via polarizing into different phenotypes, releasing cytokines, and mediating cellular communication to tune the osteoimmune microenvironment [4]. Traditionally, macrophages can be divided into two phenotypes, including pro-inflammatory M1 and anti-inflammatory M2 [5]. M1 macrophages produce pro-inflammatory cytokines like tumor necrosis factor-α and inducible nitric oxide synthase (iNOS), whereas M2 ones produce anti-inflammatory cytokines such as arginase 1 (Arg-1) and interleukin-4 [6,7]. Recently, many studies have devoted into promoting M2 polarization to create a good osteoimmune microenvironment, which has been an important immune strategy in the field of bone regeneration [[8], [9], [10]].

Autophagy is an evolutionarily conserved intracellular process for cell homeostasis maintenance, which promotes tissue regeneration by inflammation regulation, oxidative stress reduction and cell differentiation [11,12]. It has been reported that autophagy facilitates the transition from pro-inflammatory M1 to anti-inflammatory M2 macrophages, which may ultimately promote bone regeneration [[13], [14], [15]]. Various biomaterials were engineered to tune macrophage autophagy [[16], [17], [18]]. For example, a DNA nanostructure-mediated nanoplatform, incorporating spermidine and mTOR siRNA, was fabricated to enhance macrophage autophagy, ultimately treating acute lung injury [19]. Recently, the physicochemical properties of biomaterials such as morphology, size, surface nanotopography and surface composition can influence cellular autophagy [[20], [21], [22], [23]]. For example, the size of nanoparticles regulates the accumulation of autophagosomes and activation of autophagy [20]. The nanotopography surface also has an effect on autophagy activation and osteogenesis [21]. Therefore, tuning biomaterials’ physicochemical properties such as surface topography, size and chirality may be a promising approach to manipulate macrophage autophagy for bone regeneration.

Chirality, the inherent geometric physicochemical property of objects that are completely nonsuperimposable on their mirror images, is extensively observed in biomolecules such as proteins and nucleic acids [[24], [25], [26]]. These molecules exhibit stereochemistry-dependent interactions in biological processes [27]. Mimicking the advantageous chiral recognition observed in biological systems, engineered chiral nanomaterials have emerged as promising strategies for antibacterial, osteogenesis, nerve repair and immunomodulation [27,28]. Up to now, various chiral nanomaterials such as chiral nanoparticles, nanofibers, and carbon dots have been widely studied in immunomodulation, demonstrating their ability to influence immune cell activity and cytokine production in a chiral dependent manner [[29], [30], [31], [32]]. For instance, L-chiral ZnS NPs more effectively scavenged ROS in macrophages, in turn reducing the secretion of TNF-α, IL-6, and IL-1β, while upregulating IL-10 than D-ZnS NPs in inflammatory bowel disease [29]. While right-handed chiral supramolecular nanofiber of camptothecin induced superior ROS generation effectively modulate the tumor immune microenvironment and enhance cytotoxic T cell-mediated antitumor immunity [30]. Similarly, the D-carbon dot-based nanovaccine could trigger a stronger immune response, enhance BMDC maturation, promote T cell proliferation and cytokine secretion, thereby inducing significant T cell-mediated antitumor effects than the L-counterpart [31]. These studies collectively highlight the unique and powerful immunomodulatory capabilities of chiral nanomaterials, paving the way for novel applications in biomedical fields involving immune responses. Among them, chiral AuNPs have attracted particular attention due to their distinct effects on different immune cells [[33], [34], [35]]. L-chiral AuNPs demonstrate stronger immune cells activation including dendritic cells, T cells and natural killer cells, while D-chiral AuNPs also exhibit a stronger immune response in macrophages by repolarizing anti-inflammatory M2 phenotype toward a pro-inflammatory M1 phenotype [[33], [34], [35]]. Besides, L-chiral AuNPs preferentially upregulate autophagy in periodontal ligament stem cells, promoting osteogenic differentiation and bone regeneration, whereas their D-counterparts show limited effects [36]. It can be seen that chiral AuNPs hold the potential to stereoselectively modulate autophagy of macrophages, further regulating osteoimmune microenvironment.

**Scheme 1:**
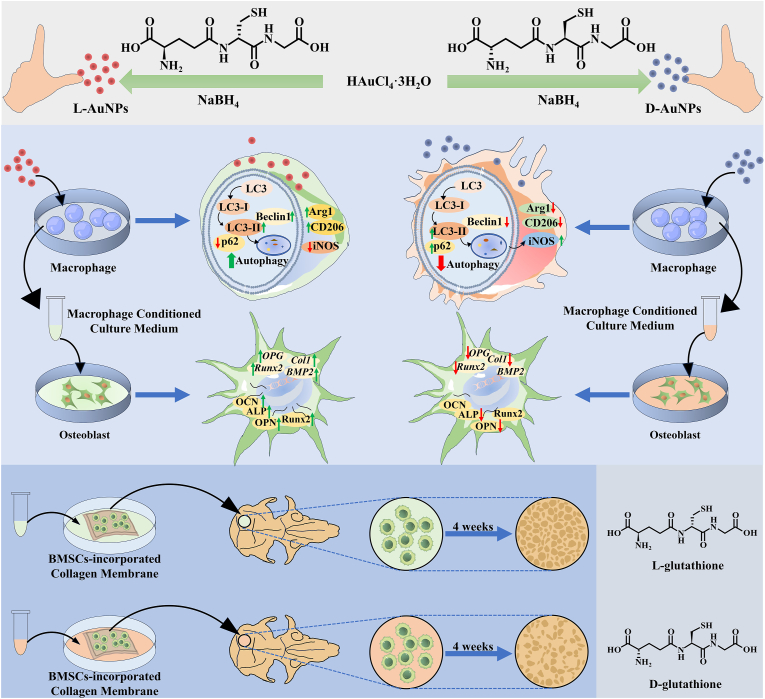
**Schematic diagram of L/D-AuNPs.** L-AuNPs exhibit enhanced macrophage uptake and increase autophagy levels, which further promotes M2 polarization and facilitates bone regeneration. In contrast, D-AuNPs suppress autophagy, tending to induce M1 polarization without significantly enhancing bone regeneration.

In this study, chiral AuNPs were synthesized with glutathione (GSH) ligands via gentle reduction of gold (III) chloride trihydrate (HAuCl_4_·3H_2_O) (Scheme 1). *In vitro* and *in vivo* results demonstrated L-AuNPs more potently activated autophagy, driving M2 macrophage polarization and fostering a pro-osteogenic immune microenvironment that enhanced bone regeneration compared to D-AuNPs. This study proved that chiral AuNPs could stereoselectively modulate autophagy of macrophages, providing an advanced strategy for bone regeneration.

## 2 Materials and methods

### 2.1 Materials

L-GSH (≥98 %) was purchased from Alfa Aesar (Shanghai, China). D-GSH (≥98 %) was bought from GL Biochem (Shanghai, China). HAuCl_4_·3H_2_O was purchased from Energy Chemical (Anhui, China). Sodium borohydride (NaBH_4_) was bought from Xilong Scientific (Guangdong, China). Primary antibody mainly included F4/80 (HuaBio, Shandong, China), inducible nitric oxide synthase (iNOS, Proteintech, Wuhan, China), mannose receptor (CD206/MRC1, Boster, Wuhan, China), Arg-1 (Proteintech, Wuhan, China), runt-related transcription factor 2 (RUNX2, Boster, Wuhan, China), osteocalcin (OCN, Affinity, Jiangsu, China), osteopontin (OPN, Affinity, Jiangsu, China), microtubule-associated protein light chain 3 (LC3, Abcam, USA), sequestosome 1 (SQSTM1/p62, Abcam, USA), Beclin1 (CST, USA), β-actin (Proteintech, Wuhan, China). Second antibody for Western blot and immunofluorescence were got from Beyotime (Shanghai, China) and Boster (Wuhan, China) respectively. BCA Protein Assay Kit was purchased from CoWin Biotech (JiangSu, China). Polyvinylidene fluoride membranes (PVDF) was bought from Sigma-Aldrich (USA). The Bio-Gide collagen membranes were purchased from Geistlich (Switzerland). APC anti-mouse CD206 antibody and PE anti-mouse CD86 antibody were purchased from BioLegend (USA).

### 2.2 Synthesis of L/D-AuNPs

L/D-AuNPs was synthesized according to the previous reference [37]. Briefly, HAuCl_4_·3H_2_O (84 μL, 476.3 mM) was mixed with fresh L- or D-GSH aqueous solution (5 mL, 24 mM), which was then poured into 36 mL H_2_O under stirring, and then NaBH_4_ aqueous solution (2 mL, 440 mM) was added and kept stirring for 2 h. Then, the product was collected via dialysis to remove free GSH molecules and then dispersed in aqueous solution.

### 2.3 Characterization

The physicochemical properties of L/D-AuNPs were thoroughly characterized using a range of analytical techniques. The zeta potential and hydrodynamic diameter were determined using a Zetasizer Nano ZS instrument (90Plus PALS, Brookhaven, USA). Morphological analysis was carried out by transmission electron microscopy (TEM, JEM2100, JEOL, Japan). To evaluate their optical characteristics, UV–visible spectroscopy was performed with a V-750 spectrophotometer (JASCO, Japan). The chemical composition and surface functional groups were analyzed using Fourier transform infrared (FTIR) spectroscopy (FT/IR-4700, JASCO, Japan). In addition, circular dichroism (CD) spectroscopy was employed to assess the optical activity of the nanoparticles (J1500, JASCO, Japan). Finally, the gold content in L/D-AuNPs was quantified via inductively coupled plasma mass spectrometry (ICP-MS, 7500 Series, Agilent, USA).

### 2.4 Cytotoxicity assay

RAW264.7 and MC3T3-E1 cells were treated with L/D-AuNPs at various concentrations of 0.5, 1, 5, 10 and 20 μM for 24 h. Then cytotoxicity of L/D-AuNPs was detected by Live-Dead staining and cytoskeleton staining with AO/EB Double Staining Kit and TRITC Phalloidin respectively. Subsequently, cells were subjected to inverted fluorescence microscopy (DMi8, Leica, Germany). Meanwhile, the cell proliferation capacity was assessed using the CCK-8 assay, with optical density (OD) measurements recorded at 450 nm on days 1 and 3, respectively.

### 2.5 Cellular uptake

To obtain L/D-AuNPs labeled by fluorescein isothiocyanate (FITC), 5 mL L/D-AuNPs was added into 5 mL FITC solution dissolved into DMSO (0.2 mg/mL) and then stirred fully followed by dialyzing to remove free FITC molecules. The L/D-AuNPs labeled by FITC was collected via centrifugation and dispersed in 5 ml of water. Then, RAW264.7 cells seeded onto confocal dishes and further incubated with L/D-AuNPs labeled by FITC (5 μM) for 3 h [38]. After staining nuclei by DAPI according to protocols, the cellular uptake was observed by confocal fluorescence microscope (Spin SR, Olympvs, Japan).

### 2.6 Western blot

To investigate the influence of L/D-AuNPs on macrophage autophagy, RAW264.7 treated with L/D-AuNPs (5 μM) for 24 h were subjected to Western blot. After the protein was transferred onto PVDF via gel electrophoresis, the membrane was sequentially incubated with primary and secondary antibodies for 12 h and 2 h, respectively. The primary antibodies were used include LC3, p62, Beclin1 and β-actin here. Finally, the expression level of various protein was observed by reacting with chemical developer and digital imaging. To investigate the influence of L/D-AuNPs on macrophage phenotype, RAW264.7 were treated with L/D-AuNPs (5 μM) for 48 h. Then, all remaining procedures followed those established for RAW264.7 above except for the primary antibody like iNOS, CD206 and Arg-1.

In order to detect the effect of macrophage pretreated by chiral AuNPs on osteogenic differentiation, RAW264.7 were cultured with L/D-AuNPs for 48 h to collect the supernatant by centrifugation and filtration. Next, macrophage-conditioned medium (CM) was prepared by mixing cell culture supernatant with osteogenic induction medium at a 2:1 volumetric ratio and used to induce MC3T3-E1 cells osteogenic differentiation and changed every 2 days. After 7 days, the cells were subjected to detect expression levels of osteoblast-related proteins by Western blot. All remaining procedures followed those established for RAW264.7 except for the primary antibody like RUNX2, OPN and OCN.

### 2.7 Immunofluorescence staining

To investigate the influence of chiral AuNPs on macrophage autophagy, RAW264.7 were treated with L/D-AuNPs (5 μM) for 24 h. After be sequentially undergoing the steps of fixation, penetration, and blocking, the cells were further incubated with LC3 and p62 primary antibody for 12 h prior to incubation with secondary antibody for 2 h and observed by inverted fluorescence microscope finally. To investigate the influence of chiral AuNPs on macrophage phenotype, RAW264.7 were treated by L/D-AuNPs (5 μM) for 48 h and then the immunofluorescence staining was carried out. All remaining procedures followed those established for RAW264.7 above except for the primary antibody like iNOS, Arg1 and CD206. In order to investigate the relationship between macrophage polarization and autophagy by L/D-AuNPs, 3-methyladenine (3-MA, 2.5 μM) was used to pre-stimulate RAW264.7 cells for 6 h to inhibit autophagy before L/D-AuNPs treatment.

### 2.8 Flow cytometry analysis

After treated with L/D-AuNPs (5 μM) for 48 h, RAW264.7 cells were washed and resuspended in 100 μL PBS containing 1 % FBS. Then, the cells were blocked and incubated with PE-CD86 or APC-CD206 antibody (0.2 μg/test) at 4 °C for 30 min in sequence. After resuspended in PBS and filtered to obtain single-cell suspension, the cells were detected by a flow cytometer (CytoFlex, Beckman, USA), and FlowJo-v10.8.1 software (Treestar, USA) was used to analyze the data.

### 2.9 Quantitative real-time polymerase chain reaction (RT-qPCR)

To detect the effect of macrophage phenotype induced by chiral AuNPs on mRNA levels of osteogenic genes, MC3T3-E1 were treated with different CMs for 7 days and then subjected to RT-qPCR. The total mRNA was obtained with trizol prior to reverse-transcription and further amplification by RT-qPCR. The 2^−ΔΔCt^ method was used to quantify the mRNA levels of osteogenic genes and primer sequences were shown in supplementary materials (Table S1).

### 2.10 Alkaline phosphatase (ALP) activity and staining

To evaluate ALP activity of MC3T3-E1 induced by chiral AuNPs, the cells were treated as described in 2.9 section. After fixation, MC3T3-E1cells were subjected to ALP staining via BCIP/NBT ALP staining kit and then observed using stereomicroscope (S9i, Leica, Germany). For quantitative analysis of ALP activity, the supernatant of MC3T3-E1 was collected after lysing and centrifuging. Then, ALP activity was assessed by the ALP Detection Kit which was normalized to the control group.

### 2.11 Alizarin red S (ARS) staining and quantification

MC3T3-E1cells were treated by different CMs as described in 2.9 section for 21 days. After staining with 2 % ARS staining solution (pH = 4.1) and air-drying, the cells were observed by a stereomicroscope. Then, 10 % (w/v) cetylpyridinium chloride was used to dissolve the stained mineral deposition and further quantification of ARS was conducted by spectrophotometric absorbance measurement at 562 nm.

### 2.12 Murine subcutaneous implantation model

All animal experimental protocols were approved by the Institutional Animal Care and Use Committee of Nanchang University and performed in strict adherence to the National Institutes of Health Guidelines for the Care and Use of Laboratory Animals. Seven-week-old female C57BL/6 J mice were purchased from Ziyuan Experimental Animal Technology Co., Ltd (Hangzhou, China). After adaptive housing, the mice were randomly divided into three groups: control group, L-AuNPs group, and D-AuNPs group. All mice were anesthetized by intraperitoneal injection of 1 % pentobarbital sodium at a dose of 40–45 mg/kg. The dorsal skin (1.5 cm × 1.5 cm) was shaved and disinfected with povidone-iodine followed by 75 % ethanol. Subsequently, L- or D-AuNPs (40 μg/mL) were injected subcutaneously into the shaved area at a dose of 2.5 mg/kg, while the control group received an equivalent volume of PBS based on body weight. Three days post-surgery, the mice were euthanized by cervical dislocation under anesthesia. The skin and subcutaneous tissues from the treated area were excised and fixed in 4 % paraformaldehyde for subsequent immunofluorescence staining.

### 2.13 Rat skull defect model

Female rats at 7 weeks old (specific-pathogen free) were purchased from Corues Biotechnology (Nanjing, China). After 1 week of adaptive feeding, the experiment was carried out according to the principles of anesthesia and sterility. The full-thickness skull defects (5 mm in diameter) were surgically created in the cranial region of the rats using a trephine under saline irrigation. After removing the craniotomy segment, the defect was randomly covered with different collagen-based BMSCs tissue-engineered composites with the cell side inward facing the intracranial. Next, suturing the in layers to close the wound and then keeping intramuscular antibiotic injection 3 days after surgery to prevent infection. The collagen membrane-BMSCs composites was prepared as previous reported [[38], [39], [40]]. Briefly, 2.5 × 10^4^ BMSCs were seeded on 5 × 5 mm Bio-Gide collagen membranes in 48-well plates. After treatment with different CM for 48 h. The whole rats are divided into three groups (N = 8 per sample group) according to the group of the composites. The remaining three rats were used as blank control (Blank group). Rat body weights were monitored at 3-day intervals. Following a 4-week experimental period, all animals were humanely euthanized, after which skull specimens were surgically harvested and immersion-fixed in 4 % paraformaldehyde.

### 2.14 Micro-computed tomography (μCT) analysis

Bone regeneration within the skull defect area was accessed via μCT (NMC-200, NEMO, USA) with X-ray acquisition parameters set at 80 kV and 60 μA. Then, the μCT images were conducted three-dimensional reconstruction and quantitative analysis of bone related parameters including bone volume (BV), tissue volume (TV), BV/TV, bone mineral density (BMD), trabecular thickness (Tb.Th), and trabecular number (Tb.N).

### 2.15 Histological staining

After blocking with 10 % normal serum, the tissue sections of subcutaneous implantation model were incubated with F4/80, CD206 and iNOS primary antibody for 12 h respectively. Then, the tissue sections were incubated with the corresponding secondary antibody for 0.5 h. After washing, an inverted fluorescence microscope was used to image the sections.

For rat skull defect model, all skull specimens were decalcified using a solution of ethylene diamine tetra acetic acid (EDTA), which was changed every 2 days. When a syringe could penetrate the bone tissues easily, it was the time to finish decalcification. After embedding the skulls with paraffin, a microtome was used to section them a thickness of 3–4 μm. Next, the skull sections via Hematoxylin and Eosin staining (H&E) and Masson staining were imaged by stereomicroscope. For important organs, paraffin embedding and subsequent operations were performed without decalcification, and the other steps were the same as the skull specimens. After blocking with 10 % normal sera, the skull sections were incubated with RUNX2, OPN and OCN primary antibody for 12 h and then kept incubation with the secondary antibody for 0.5 h. Lastly, the inverted fluorescence microscope was used to image the skull sections.

### 2.16 Statistical analysis

The statistical analysis program SPSS 25.0 was used to examine all of the data, which were given with the mean ± standard deviation. A two-tailed Student's t-test was used to compare the two experimental groups statistically. Tukey's post hoc test was utilized in conjunction with one-way ANOVA to examine differences between three or more groups (∗∗∗P < 0.001, ∗∗P < 0.01, ∗P < 0.05).

## 3 Results and discussion

### 3.1 Characterization of chiral AuNPs

**Fig. 1:**
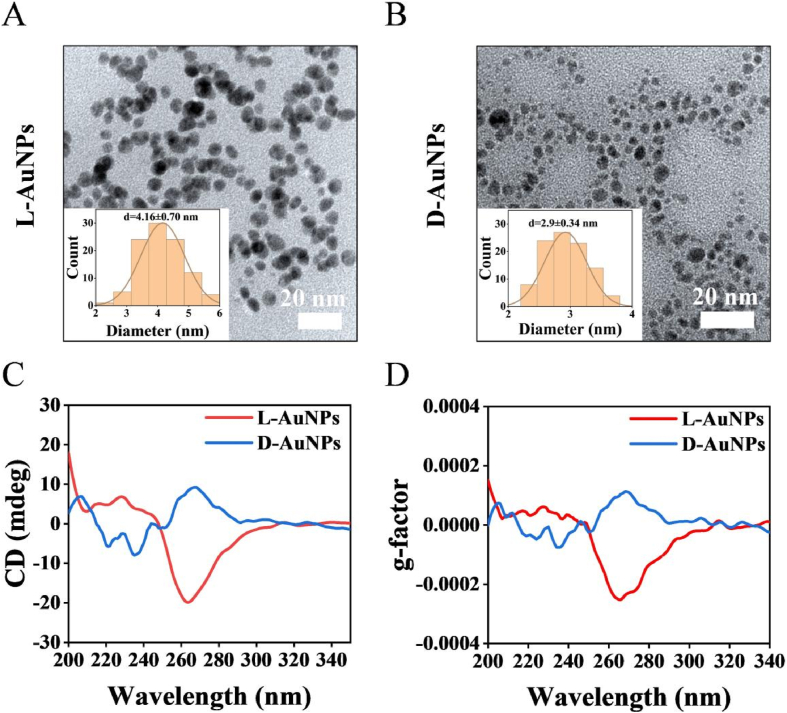
**Characterization of L/D-AuNPs.** (A, B) TEM images of L/D-AuNPs, the inset exhibits size distribution histograms of L/D-AuNPs (n = 100 nanoparticles). (C, D) CD spectra (C) and g-factor (D) of L/D-AuNPs.

Both L- and D-AuNPs could be well dispersed in the aqueous solution (Fig. S1A). The TEM images revealed that chiral AuNPs were highly uniform with narrow size distribution. L-AuNPs and D-AuNPs were roughly 4.16 ± 0.70 and 2.9 ± 0.34 nm in size, respectively (Fig. 1A and B). The hydrodynamic diameter of L-AuNPs were also slightly larger than that of D-AuNPs (Fig. S1B and C). L-AuNPs possessed a characteristic UV–vis absorption peak at about 550 nm while the typical surface plasmon resonance bands of D-AuNPs were not observed, which may be caused by their small size [37] (Fig. S1D). The zeta potential values of two kinds of AuNPs were similar with negative values (Fig. S1E). FTIR spectroscopy showed that the absorption peak disappeared at about 2500 cm^−1^ (Fig. S1F), indicated that the chiral GSH molecules were reacted with gold via Au-S bonds. The CD spectrum shapes of L-GSH and L-AuNPs were similar, and a dramatic red shift for L-AuNPs was observed in the CD spectrum with peak at around 260 nm in comparison with the L-GSH, and this phenomenon also appeared in D-AuNPs (Fig. 1C, D, Fig. S2A and B). These CD spectra and g-factor of L/D-AuNPs mirrored identically in the same region (Fig. 1C, D, Fig. S2A and B). The above results demonstrated the successful modification of chiral ligands with opposite chirality [41]. After 1 week storage in H_2_O, the CD signal and g-factor of L/D-AuNPs were similar with those observed a week ago, indicating excellent stability of L/D-AuNPs, which would benefit their biological applications (Fig. 1C, D, Fig. S2C–F). The results above proved the successful synthesis and the stability of the chiral AuNPs.

### 3.2 Macrophage polarization regulated by chiral AuNPs via autophagy

Considering that biocompatibility was a prerequisite for gold nanoparticles, the potential toxicity of L/D-AuNPs was investigated firstly. By Live-Dead staining, no significant cell death was observed after L-AuNPs or D-AuNPs treatment (Figs. S3 and S4). The cell morphology of L/D-AuNPs groups was similar with that of the control via cytoskeleton staining (Figs. S3 and S4). Furthermore, the results of CCK-8 showed that L/D-AuNPs exhibited no significant inhibitory effects on cell proliferation (Fig. S5). These results indicated desirable cytocompatibility of L/D-AuNPs.

**Fig. 2:**
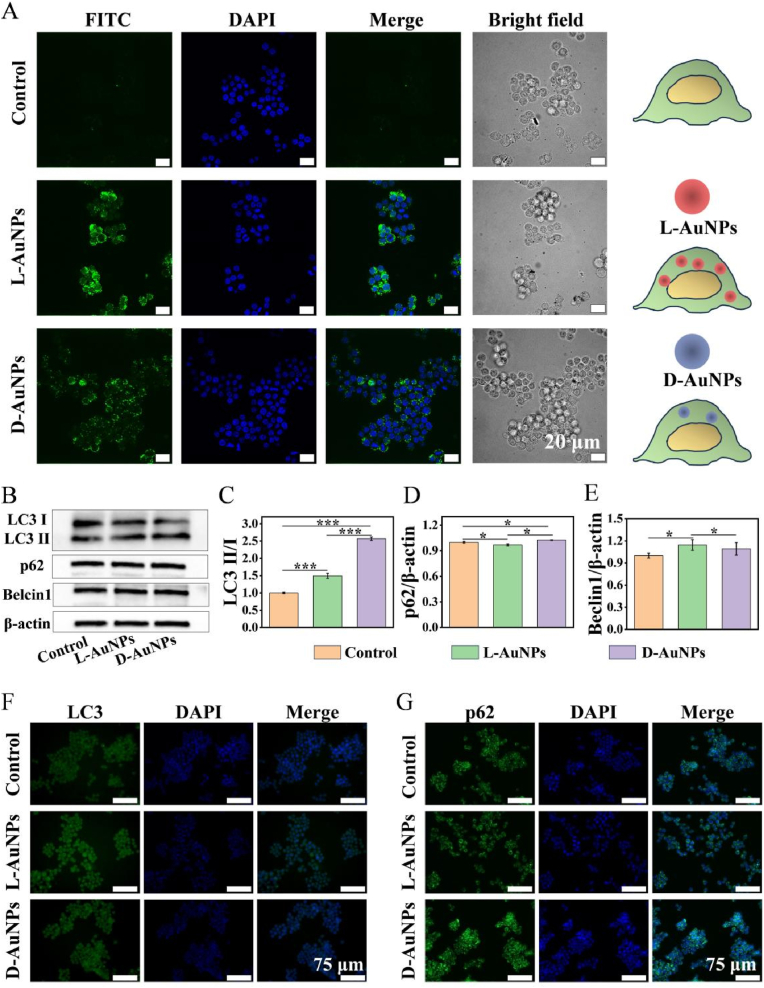
**Cellular uptake and autophagy of macrophage with L/D-AuNPs treatment.** (A) Confocal images of RAW264.7 after treatment with L/D-AuNPs (5 μM) for 3 h (L/D-AuNPs: green; nuclear: blue). (B–E) Western blot analysis of Beclin1, p62 and LC3II/I expression in RAW264.7 after treatment with L/D-AuNPs. (F, G) Immunofluorescent images of RAW264.7 in each group (LC3/p62: green; nuclear: blue). All data were presented as the means ± SD (n = 3). ∗p < 0.05, ∗∗p < 0.01, and ∗∗∗p < 0.001.

For many nanoparticles (NPs), their safe entry into cells is an important step in to exert biological effects [42]. Hence, cellular uptake of L/D-AuNPs were observed by confocal microscopy. The macrophages treated with L-AuNPs showed stronger fluorescence than those treated with D-AuNPs (Fig. 2A), implying that chirality is able to affect the macrophages internalization of nanoparticles. Left-handed chirality may contribute to enhanced cellular uptake of L-AuNPs by coordinating cell-material adhesion through the transmembrane mechanoreceptor integrin β1 and FAK [43]. In addition, previous studies have indicated that L-phospholipids constituted the primary constituents of cell membranes, making L-AuNPs easier to being taken up by macrophages than D-AuNPs [41]. Meanwhile, L-AuNPs exhibited higher affinity for adhesion G protein-coupled receptor CD97 and EMR1 on immune cell membranes than D-AuNPs, triggering clathrin-mediated endocytosis to increase cellular internalization [33]. Such observed differential uptake efficiency of L-AuNPs and D-AuNPs by macrophages likely influenced subsequent biological processes, a pattern that extended to affect macrophage autophagy. The result of Western blot analysis demonstrated significantly elevated LC3-II/I ratios in the D-AuNPs-treated group relative to the L-AuNPs group. However, autophagy was a dynamic process so that the expression level of LC3II/I at a single time point could not accurately explain the change of autophagy [44,45]. During autophagosome formation, p62 could connected LC3 and polyubiquitination protein and further selectively wrapped into the autophagosome followed by degradation via enzymolysis in the autolysosome [46]. The expression level of p62 in macrophages was reduced by L-AuNPs but upregulated by D-AuNPs (Fig. 2B–D). Up-regulation of LC3II/I accompanied with down-regulation of p62 in L-AuNPs group suggested the promotion of autophagy in macrophages. Simultaneous upregulation of LC3II/I and p62 in D-AuNPs indicated inhibition of autophagy [47]. Furthermore, Beclin1 as a positive regulatory factor of autophagy was detected, which mediated the assembly of autophagy-related complex through interactions with other core proteins [48]. L-AuNPs enhanced the expression of Belcin1 while no obvious change in D-AuNPs group was observed, proving chirality-dependent regulatory effects of AuNPs on macrophage autophagy (Fig. 2B–E). Moreover, immunofluorescence intuitively revealed that brighter fluorescence of LC3 with dimmer fluorescence of p62 in L-AuNPs treatment group, while the D-AuNPs treatment group presented a completely opposite trend (Fig. 2F and G). This was consistent with the results of Western blot, demonstrating that L-AuNPs appeared to promote macrophage autophagy whereas D-AuNPs inhibited it.

**Fig. 3:**
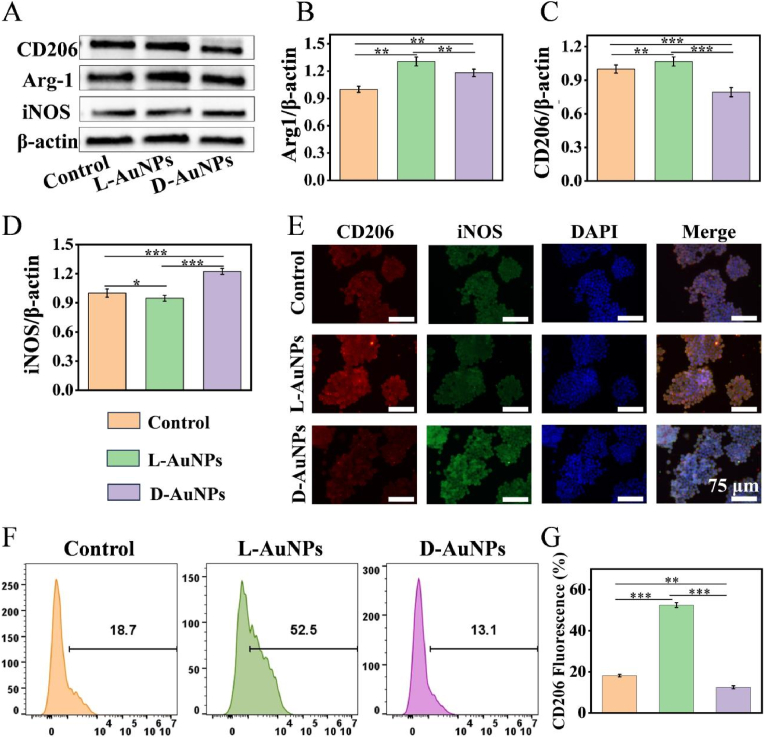
**L/D-AuNPs regulated macrophage polarization.** (A–D) Western blot analysis of CD206, Arg1 and iNOS expression in RAW264.7 after treatment with L/D-AuNPs for 48 h. (E) Immunofluorescent images of RAW264.7 cells treated by L/D-AuNPs for 48 h (iNOS: green; CD206: red; nuclear: blue). (F, G) Flow cytometry analysis of CD206 expression in RAW264.7 cells after L/D-AuNPs treatments for 48 h. All data were presented as the means ± SD (n = 3). ∗p < 0.05, ∗∗p < 0.01, and ∗∗∗p < 0.001.

Autophagy played a significant regulatory role in macrophage polarization [[49], [50], [51]]. Inspired by chiral selectivity of autophagy based on above results (Fig. 2B–G), the modulation of L/D-AuNPs on macrophage phenotypes were further investigated. After the cells cultured with L/D-AuNPs, the protein expression of M2 marker (CD206 and Arg-1) and M1 marker (iNOS) was evaluated by Western blot. L-AuNPs facilitated the generation of CD206 and Arg1 while decreased the formation of iNOS (Fig. 3A–D). However, D-AuNPs aggravated the expression of iNOS and inhibited the formation of CD206, even though somewhat upregulated Arg1 expression (Fig. 3A–D). These results implied that there was a distinct effect in macrophage phenotypes by L/D-AuNPs. Meanwhile, immunofluorescence staining showed that the cells treated with L-AuNPs displayed more stronger fluorescence intensity in CD206 and Arg-1 but weaker in iNOS than D-AuNPs (Fig. 3E–S6), also confirming that chiral AuNPs induced differential macrophage polarization. Moreover, the results of flow cytometry analysis revealed that L-AuNPs significantly increased the proportion of CD206^+^ cells, whereas D-AuNPs decreased it, with no obvious difference in CD86^+^ cell proportion between L-AuNPs and D-AuNPs (Fig. 3F, G, S7). Collectively, these results indicate that L/D-AuNPs modulate macrophage phenotypes in a chirality-dependent manner, with L-AuNPs promoting M2 polarization and D-AuNPs exhibiting a tendency toward M1 polarization.

**Fig. 4:**
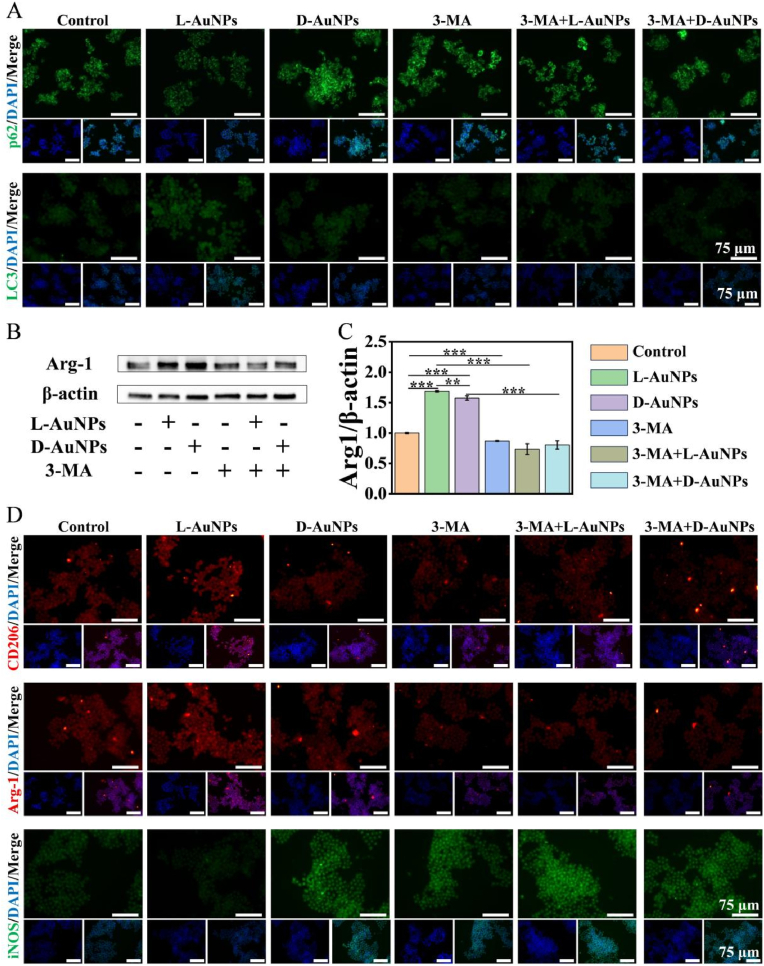
**L/D-AuNPs regulated macrophage polarization via autophagy.** RAW264.7 cells with or without 3-MA (2.5 μM) pretreatment for 6 h. (A) Immunofluorescent images of LC3/p62: green; nuclear: blue. (B, C) Western blot images and quantitative analysis of Arg-1 expression. (D) Immunofluorescent images of iNOS: green; CD206/Arg-1: red; nuclear: blue. All data were presented as the means ± SD (n = 3). ∗p < 0.05, ∗∗p < 0.01, and ∗∗∗p < 0.001.

To further investigate the link between autophagy and macrophage phenotype by chiral AuNPs, 3-MA as autophagy inhibitor was used to pre-stimulate macrophages before L/D-AuNPs treatment. In the presence of the inhibitor, the fluorescence of LC3 became dimmer whereas fluorescence of p62 became brighter, making the autophagy difference among groups attenuated or even disappeared (Fig. 4A). Simultaneously, the expression of Arg1 in macrophages treated with L/D-AuNPs were comparable to the control following 3-MA pretreatment (Fig. 4B and C), suggesting chiral-AuNPs could induce differential autophagy to further modulate the macrophage polarization. Consistently, the immunofluorescent change of CD206, Arg-1 and iNOS induced with L/D-AuNPs vanished by 3-MA pretreatment (Fig. 4D). These results suggested the distinct regulation of macrophage phenotype by L/D-AuNPs was closely related to chiral dependent autophagy manipulation.

### 3.3 Osteogenic properties in vitro

**Fig. 5:**
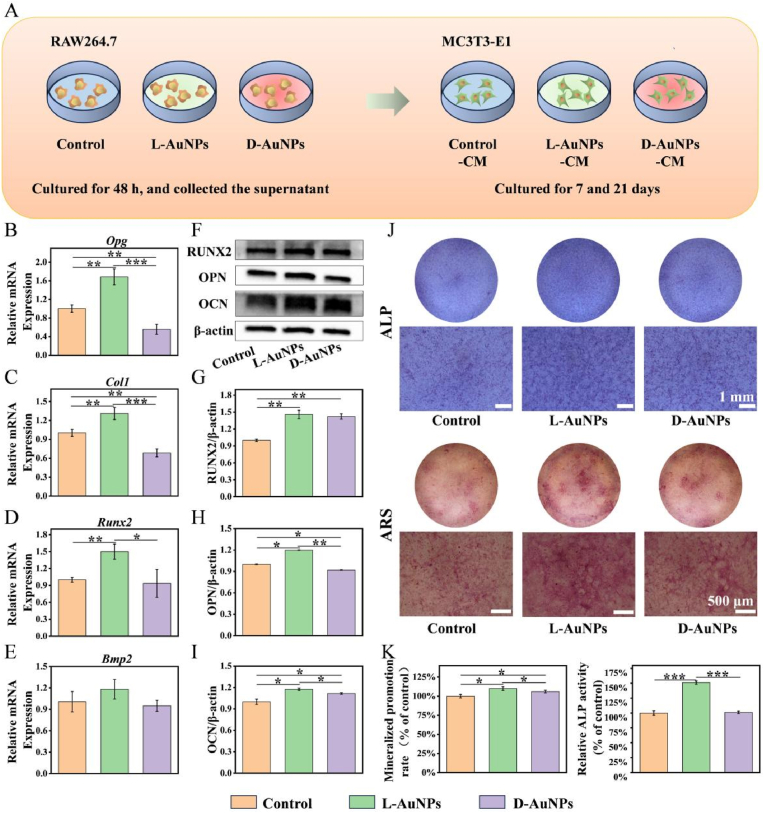
**Chiral AuNPs regulated osteogenic differentiation via macrophage polarization *in vitro*.** (A) Schematic representation of several macrophage-CMs for osteogenic induction of MC3T3-E1. (B–E) The relative mRNA levels of osteogenesis genes of MC3T3-E1 treated with CMs after 7 days. (F–I) Western blot analysis of OPN, OCN and RUNX2 expression in MC3T3-E1 after treatment with CMs after 7 days. (J) ALP staining photos of MC3T3-E1 treated with CMs after 7 days, followed by ARS staining after 21 days. (K) Quantitative assessment of ALP activity and mineralization *in vitro*. All data were presented as the means ± SD (n = 3). ∗p < 0.05, ∗∗p < 0.01, and ∗∗∗p < 0.001.

To study the immune modulatory effects of L/D-AuNPs on osteogenesis, osteoblasts’ osteogenic capability was tested in macrophage-CMs using the appropriate methods illustrated in the schematic picture. (Fig. 5A). After 7 days, the cells were subjected to RT-qPCR assay, Western blot, immunofluorescence, ALP staining and semiquantitative analysis, respectively. Compared to the other two groups, osteogenesis-related genes including *Opg*, *Runx-2*, *Bmp2* and *Col-1* all displayed up-regulation in the L-AuNPs group (Fig. 5B–E). Consistently, the protein expression of RUNX2, OPN and OCN were all remarkably increased by L-AuNPs through Western blot (Fig. 5F–I). These results indicated L-AuNPs better promoted osteoblasts osteogenic differentiation by regulating macrophage polarization. Similarly, the L-AuNPs-CM also enhanced the ALP activity via ALP staining and semiquantitative analysis (Fig. 5J and K). After 21 days, ARS staining and semiquantitative results demonstrated that the formation of mineralized nodules in osteoblasts was effectively facilitated in L-AuNPs group (Fig. 5J and K). Hence, these results implied that the immune environment induced by L-AuNPs was more favorable to the osteogenic differentiation and calcium deposition than D-AuNPs *in vitro*, demonstrating that L-AuNPs possessed outstanding potential and utility for bone regeneration.

### 3.4 Bone regeneration potential in vivo

The assessment of the biological safety of chiral AuNPs is of paramount importance prior to their application in bone regeneration; therefore, it was evaluated through *in vivo* experiments. The body weights of rats were not obviously affected by L/D-AuNPs (Fig. S8). Consistently, all groups had no obvious pathological change in the organs by H&E staining (Fig. S9), indicating L/D-AuNPs exhibited a good biocompatibility *in vivo*. Previous studies have shown that the biological toxicity of NPs is size-dependent. NPs smaller than 6 nm will be filtered through the kidneys and excreted in the urine, while NPs larger than 6 nm will accumulate in the liver over the long term, particularly those exceeding 100 nm [52,53]. Given the ultra-small size (<6 nm) of L/D-AuNPs in this study (Fig. 1A and B), they may be efficiently excreted via the renal pathway, thereby reducing organ accumulation and toxicity, which further demonstrates favorable safety *in vivo*.

To evaluate the *in vivo* immunomodulatory effects of chiral AuNPs, a murine subcutaneous implantation model was established. L-AuNPs significantly increased CD206 and decreased iNOS compared with D-AuNPs, demonstrating the ability of L-AuNPs to induce M2 macrophage polarization (Fig. S10). These results demonstrated that chiral AuNPs can elicit differential immune responses *in vivo*, which may influence subsequent osteogenic processes.

**Fig. 6:**
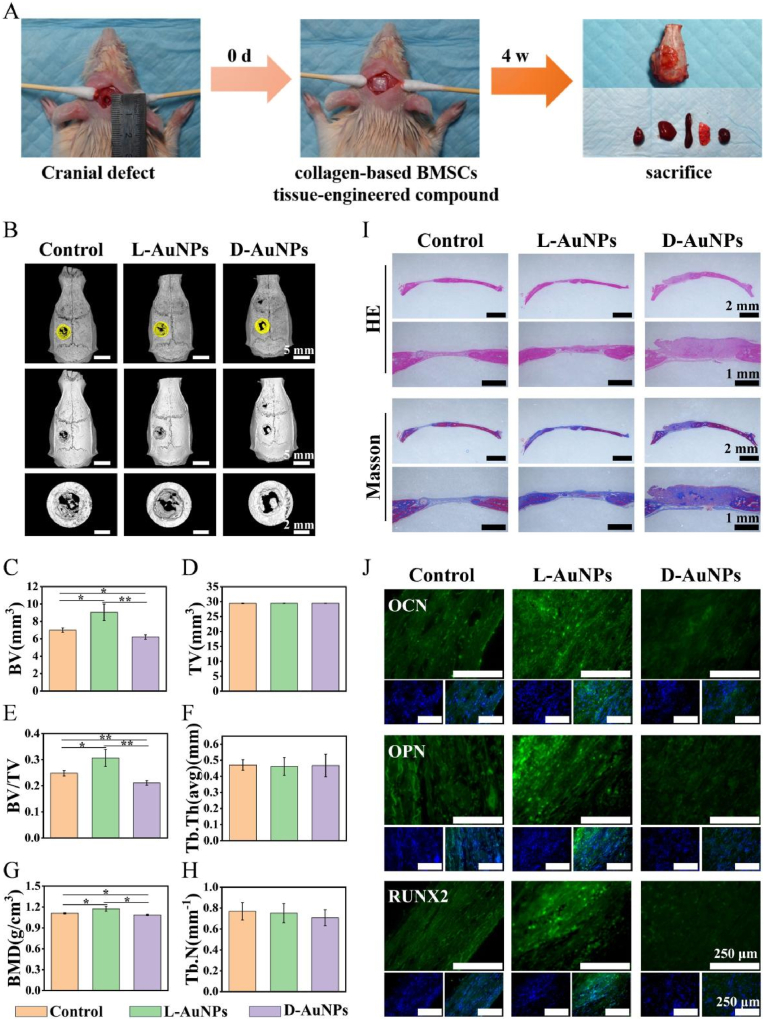
**Bone regeneration potential of chiral AuNPs *in vivo*.** (A) Schematic diagram of detection bone regeneration potential of chiral AuNPs by the rat skull defect model. (B) Micro-CT and 3D reconstructed images for skull defects. (C–H) Quantitative analysis of bone-related parameters. (I) Representative HE, Masson and (J) osteogenic-relative protein immunofluorescent staining images of skull defects (OCN, OPN and RUNX2: green; nucleus: blue). All data were presented as the means ± SD (n = 3). ∗p < 0.05, ∗∗p < 0.01, and ∗∗∗p < 0.001.

To further investigate the osteoimmune modulatory differences between L-AuNPs and D-AuNPs *in vivo*, a model of rat skull defects was utilized [39]. Onto a resorbable collagen membrane, BMSCs were sown and treated by different macrophage-CMs of L/D-AuNPs for 2 days. Then the BMSCs-membrane composites were implanted into the rat skull defect for 4 weeks as shown in the diagram (Fig. 6A). The Micro-CT results showed that there was a better capacity for bone regeneration in L-AuNPs group than that in D-AuNPs group (Fig. 6B). Meanwhile, the bone-related parameters including BV, BV/TV and BMD in L-AuNPs group also obviously increased (Fig. 6C–H), consistent with the other findings [36,54]. Furthermore, greater amounts of newly-formed bone with denser and thicker collagen fibers were found in L-AuNPs group via H&E and Masson staining (Fig. 6I). Moreover, the fluorescence intensity of OPN, OCN and RUNX2 became significantly stronger by L-AuNPs than D-AuNPs (Fig. 6J), further confirming that the L-AuNPs presented great potential of *in vivo* bone regeneration. However, the osteogenic impact of D-AuNPs *in vivo* was slightly less than that of the control, which may be attributed to the inhibition of macrophage autophagy and thus the induction of M1 polarization (Fig. 2, Fig. 3). These results suggested the osteoimmune microenvironment modulated by chiral AuNPs made distinct bone regeneration *in vivo*, showing that L-AuNPs displayed an osteogenic advantage. Besides, some published works also demonstrated that chiral AuNPs could promote osteogenic differentiation of stem cells [36,55]. However, compared with the widely used bone substitute materials such as tricalcium phosphate and hydroxyapatite, the osteogenic differentiation of chiral Au is still not enough [56,57]. As a matter of fact, the osteogenic properties of chiral Au could be improved by combining osteogenic-related cytokines such as BMP2, VEGF and RUNX2 showed excellent osteogenic abilities, theses with chiral AuNPs for bone regeneration [58]. Furthermore, these chiral AuNPs can be incorporated into bone substitute materials to enhance their immunomodulatory properties and promote osteogenic differentiation potential.

## 4 Conclusion

In conclusion, chiral AuNPs exhibit stereoselective autophagy activation in macrophages, regulating their phenotypes and subsequently affecting osteogenesis during bone regeneration. Based on chirality-dependent advantage in endocytosis, L-AuNPs promote autophagy upregulation to induce M2 macrophage polarization, thereby establishing a favorable osteogenic immune microenvironment. These findings suggest that L-AuNPs can be a promising candidate for bone regeneration. Furthermore, our work shed new light on the development of chiral nanomedicines as effective autophagy and immunological modulators for disease treatment.

## CRediT authorship contribution statement

**Jiaolong Wang:** Writing – review & editing, Methodology, Investigation, Funding acquisition. **Ning Gao:** Writing – original draft, Methodology, Investigation. **Junchao Wei:** Writing – review & editing, Funding acquisition, Conceptualization. **Lan Liao:** Writing – review & editing, Supervision, Funding acquisition, Conceptualization.

## Declaration of competing interest

The authors declare that they have no known competing financial interests or personal relationships that could have appeared to influence the work reported in this paper.

## Data Availability

Data will be made available on request.
