# Supplementary material for: Chiral gold nanoparticles manipulate osteoimmune microenvironment via macrophage autophagy for bone regeneration
Source: Mater Today Bio. 2025 Jul 25;34:102131. doi: 10.1016/j.mtbio.2025.102131 (PMC12332920; doi:10.1016/j.mtbio.2025.102131)
Supplement: Multimedia component 1 [file mmc1.docx]

**Supplementary information**


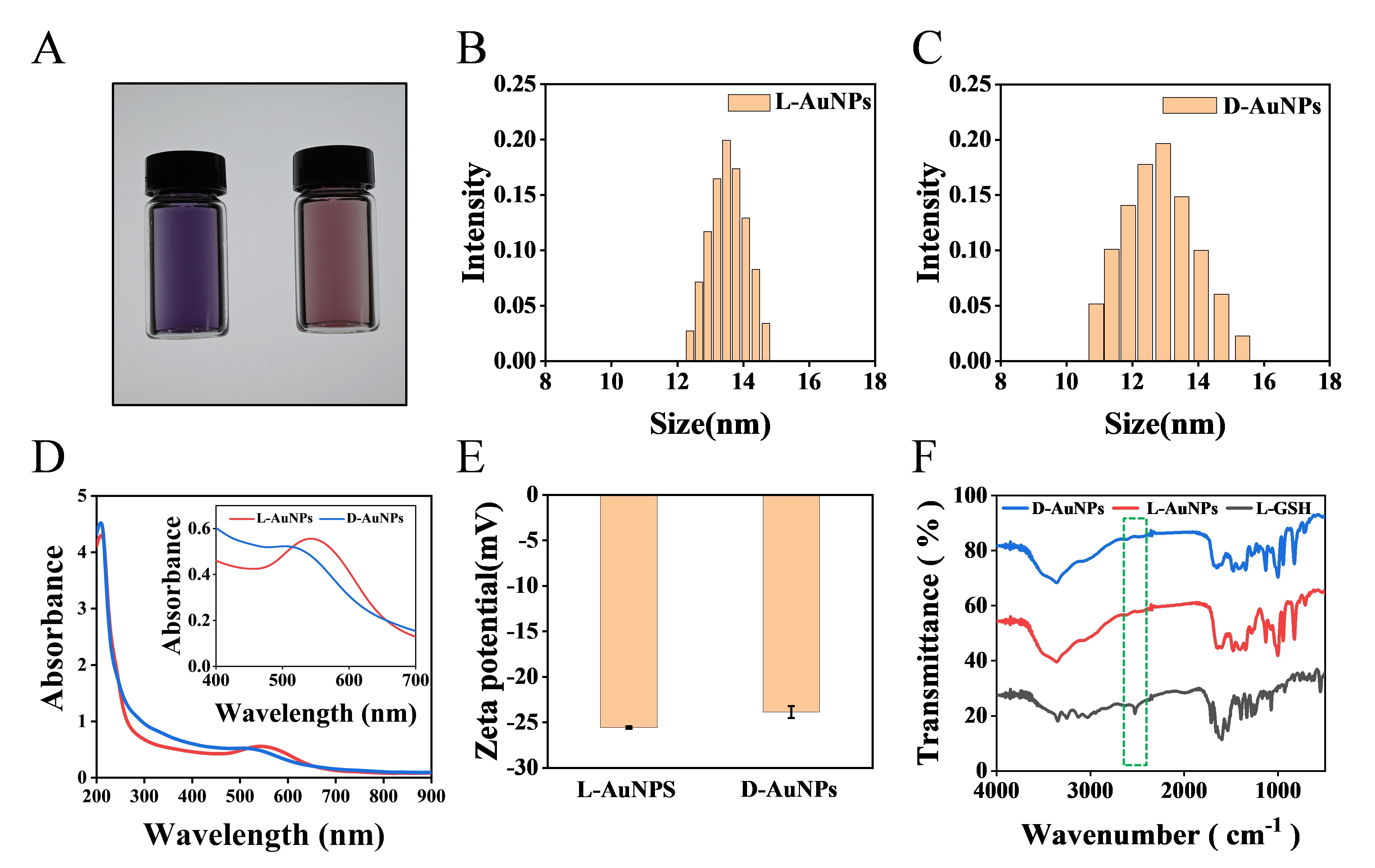


**Fig. S1.** Structure and surface characterization of L/D-AuNPs. (A) Images of L-AuNPs (left) and D-AuNPs (right). (B, C) hydrodynamic diameters of L/D-AuNPs. (D) the UV-vis absorption spectra of L/D-AuNPs. (E) zeta potential values of L/D-AuNPs. (F) FTIR spectra of L-GSH molecule, L-AuNPs and D-AuNPs. All data were presented as the means ± SD (n =3).


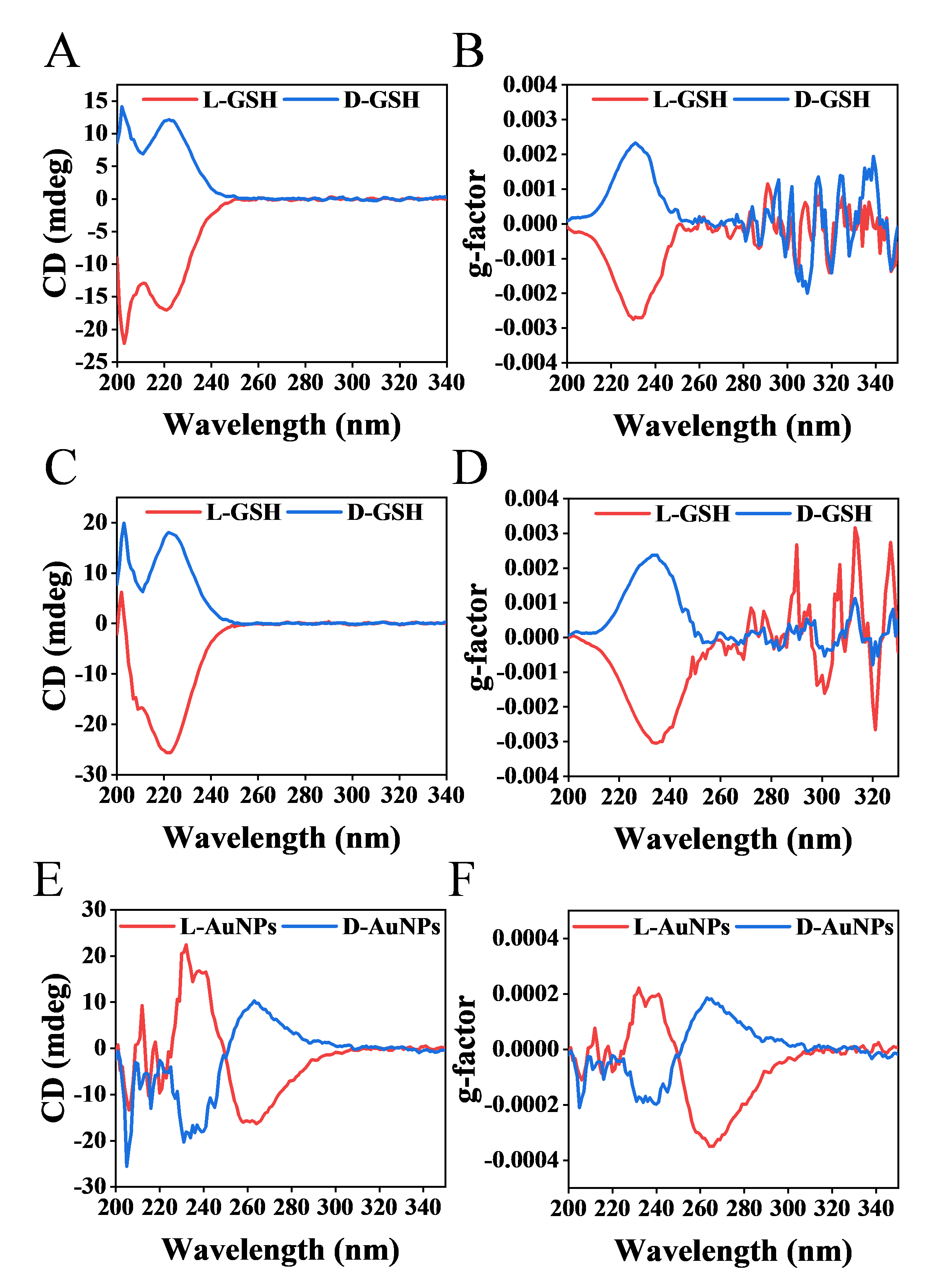


**Fig. S2.** Stability of L/D-AuNPs in water. (A-B) CD spectra and g-factor of L/D-GSH. (C-F) CD spectra and g-factor of L/D-GSH and L/D-AuNPs after being dispersed in water for 1 week.


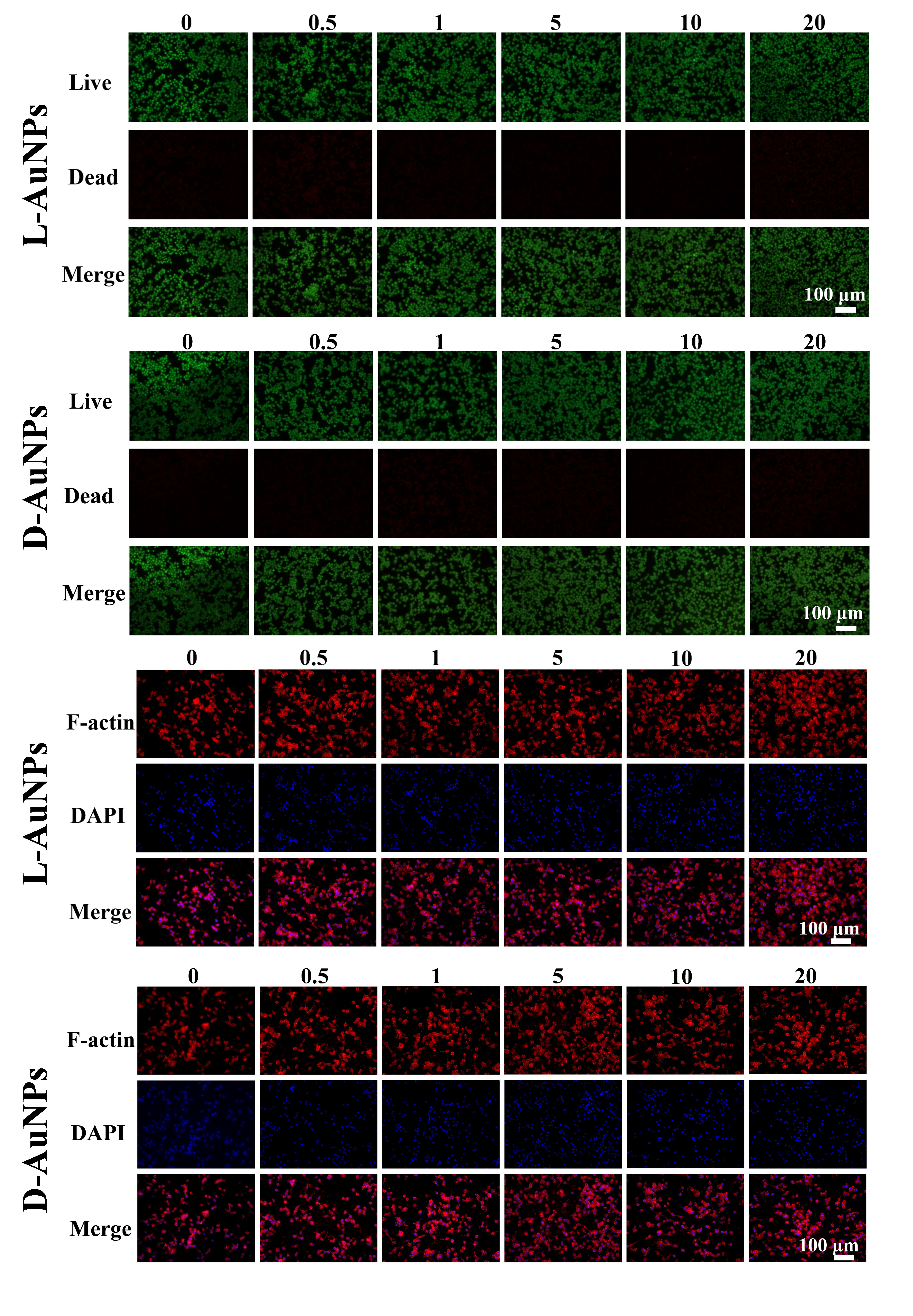


**Fig. S3.** Live-Dead staining and cytoskeleton staining of RAW264.7 cells after incubation with L/D-AuNPs at the Au concentration of 0-20 μM for 24 h (n = 3).


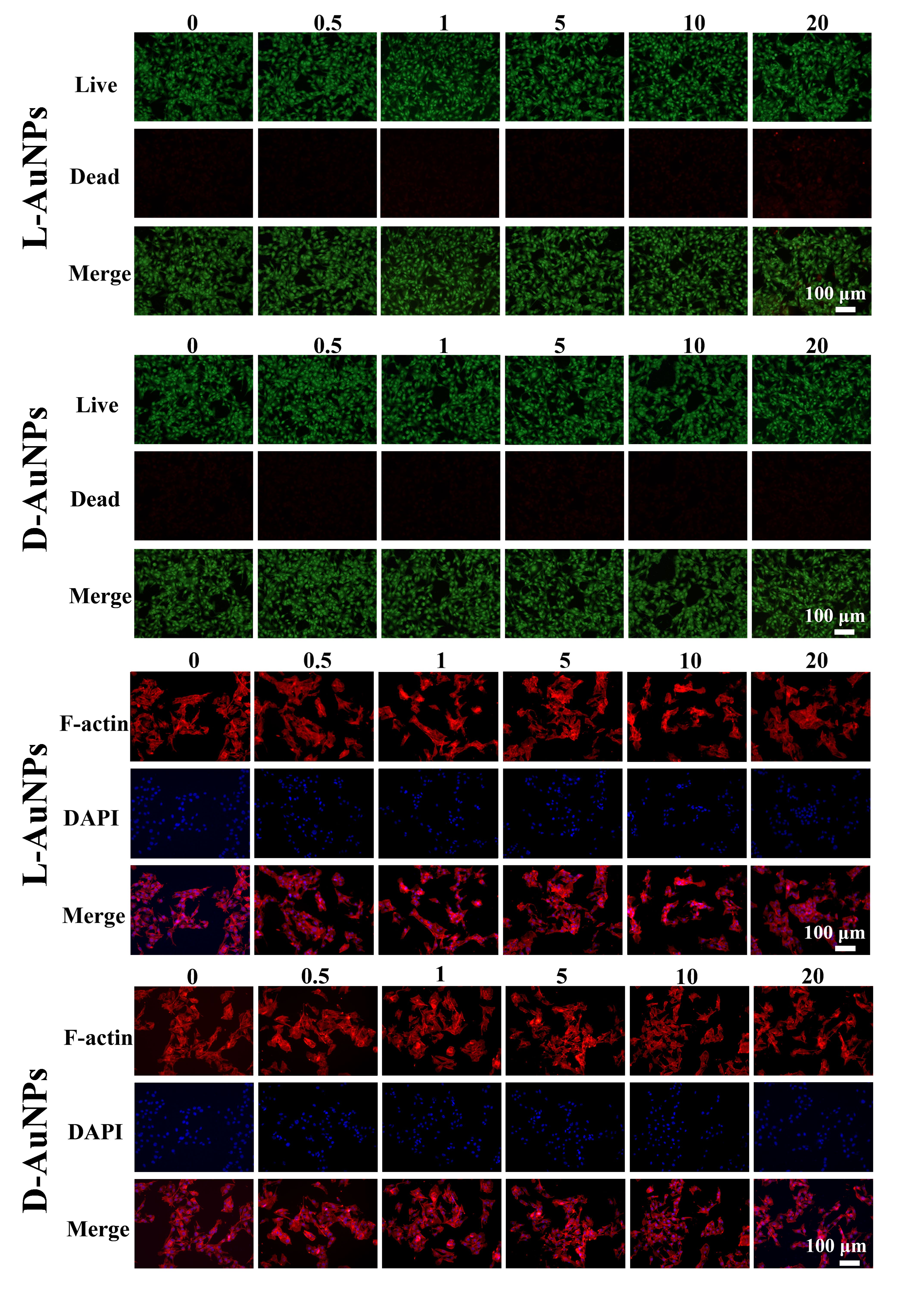


**Fig. S4.** Live-Dead staining and cytoskeleton staining of MC3T3-E1 cells after incubation with L/D-AuNPs at the Au concentration of 0-20 μM for 24 h (n = 3).


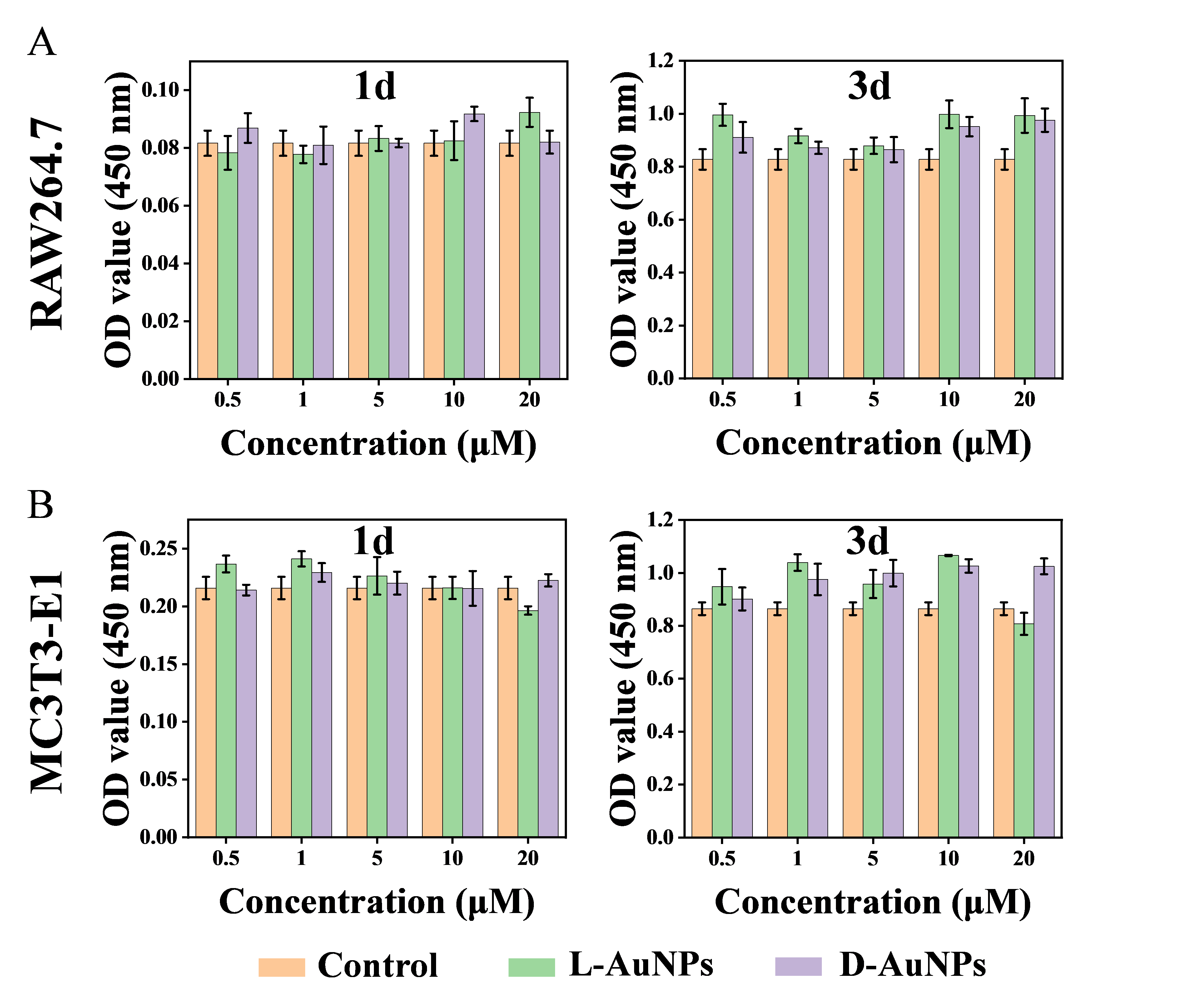


**Fig. S5.** The proliferation of RAW264.7 and MC3T3T-E1 cells after incubation with L/D-AuNPs for 1 and 3 days by the CCK-8 assay. All data were presented as the means ± SD (n =4).


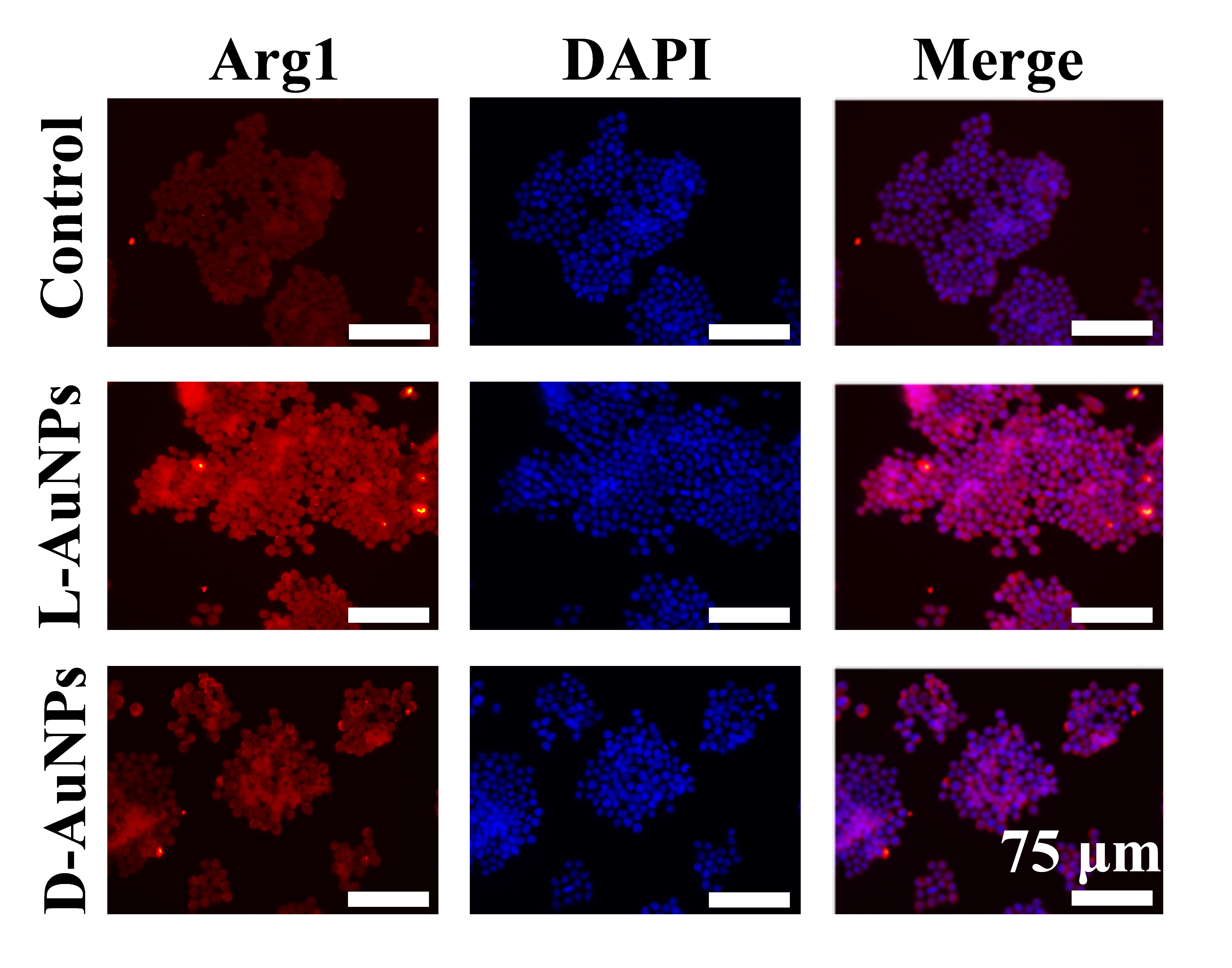


**Fig. S6.** Immunofluorescent images of RAW264.7 cells treated by L/D-AuNPs for 48 h (Arg1: red; nuclear: blue).


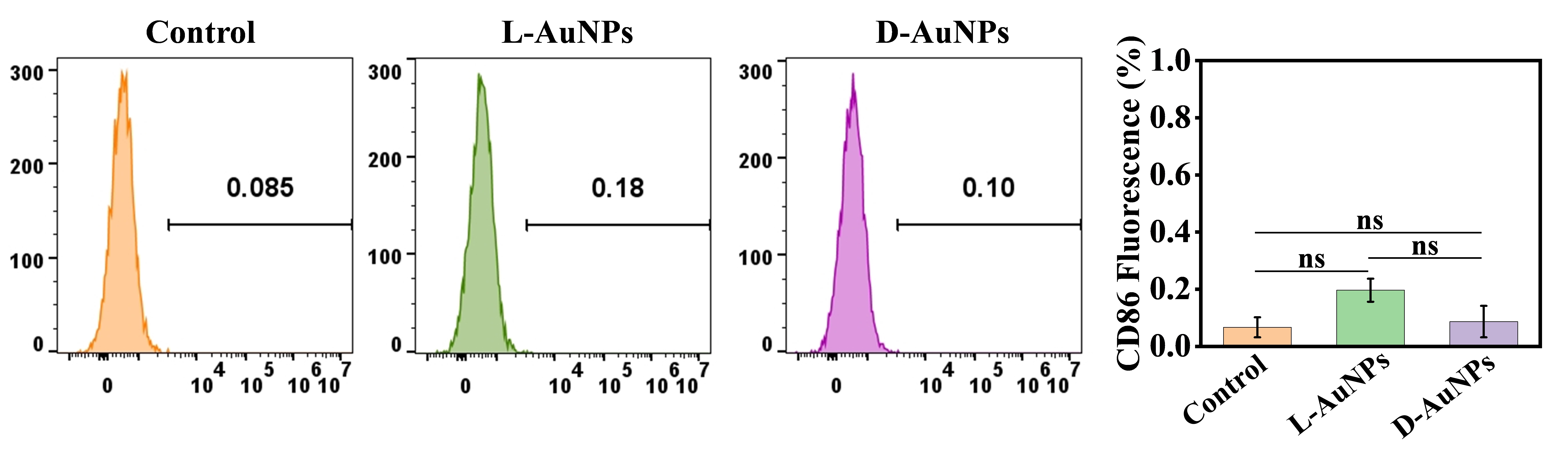


**Fig. S7.** Flow cytometry analysis of CD86 expression in RAW264.7 cells after L/D-AuNPs treatments for 48 h. All data were presented as the means ± SD (n =3). *p < 0.05, **p < 0.01, ***p < 0.001.


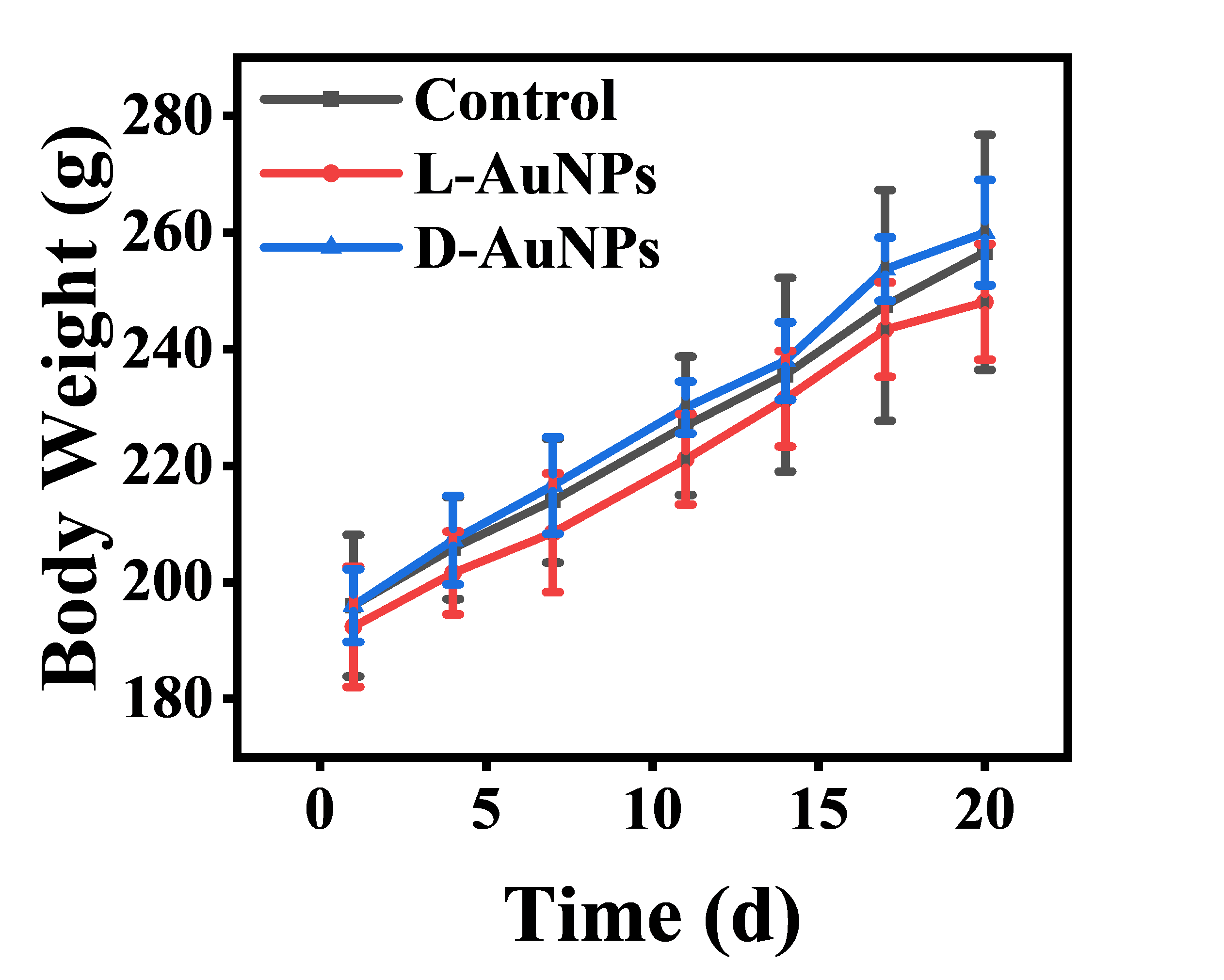


**Fig. S8.** Relative body weights of rats receiving different treatments. All data were presented as the means ± SD (n =8).


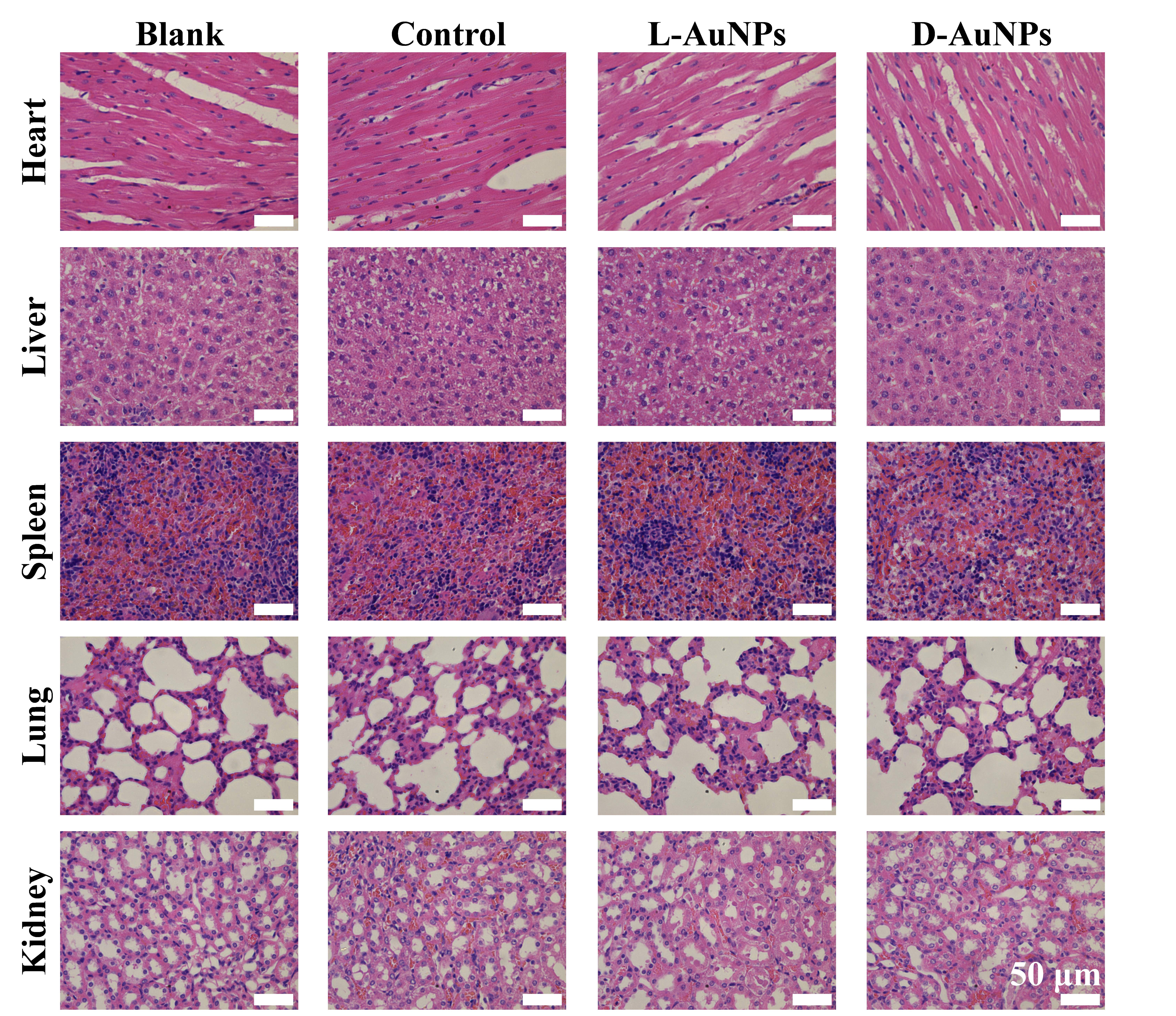


**Fig. S9.** H&E staining of the major organs after L/D-AuNPs implantation *in vivo* (n=3).


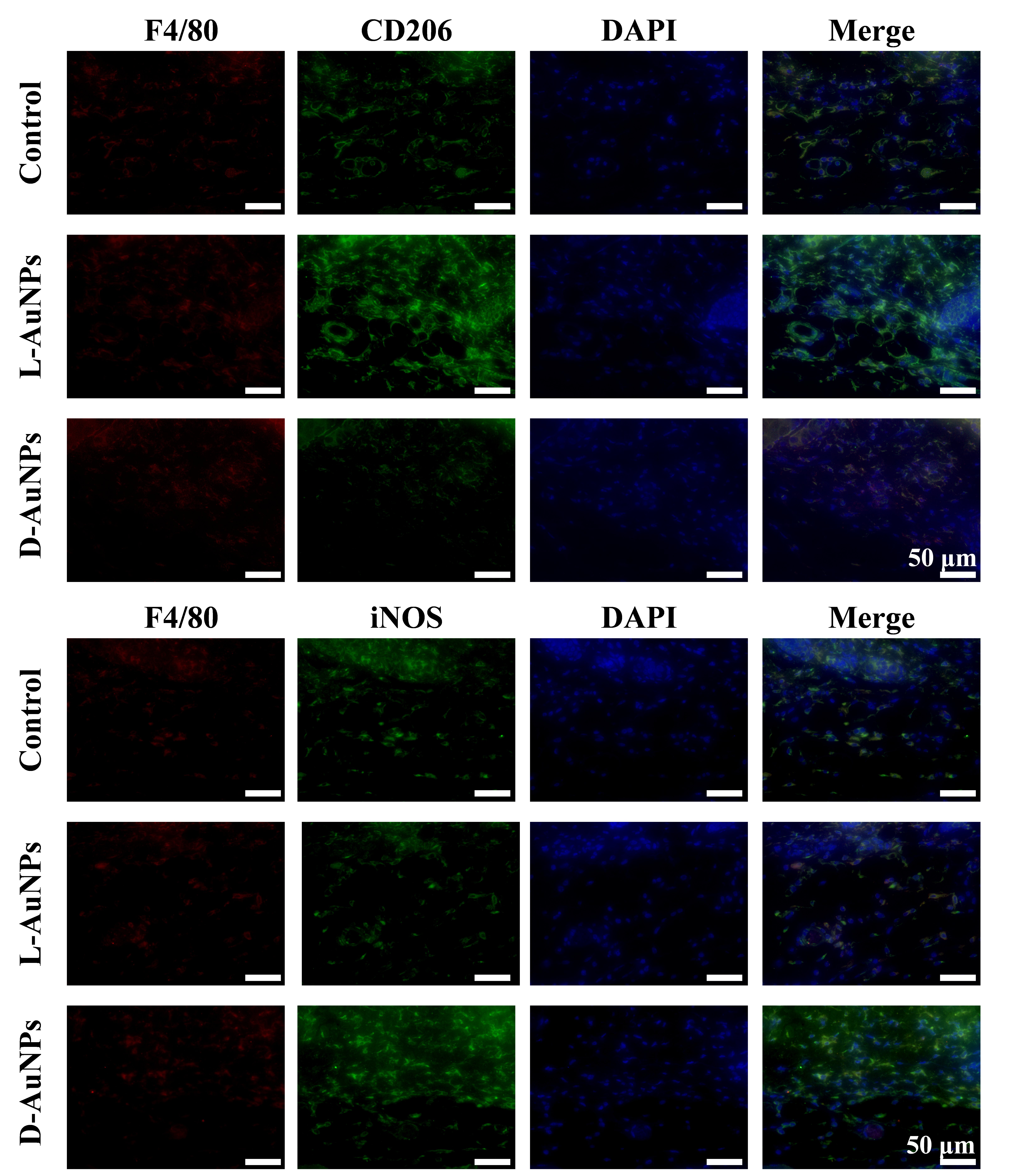


**Fig. S10.** Immunofluorescence staining of macrophages in murine subcutaneous implantation model treated by L/D-AuNPs. (F4/80: red; CD206/iNOS: green; nuclear: blue).

**Table S1.** Osteogenesis related gene primer pairs used in the RT-qPCR

| Genes | Primer sequences |
| --- | --- |
| *Gapdh* | Forward:5'-CTCCCACTCTTCCACCTTCG-3’  Reverse:5'-TTGCTGTAGCCGTATTCATT -3’ |
| *Runx2* | Forward:5'-CACTGGCGGTGCAACAAGA-3'  Reverse:5'-TTTCATAACAGCGGAGGCATTTC-3' |
| *Bmp2* | Forward:5'-GCTCCACAAACGAGAAAAGC-3’  Reverse:5'-AGCAAGGGGAAAAGGACACT-3' |
| *Opg* | Forward:5'-CGCTCGTGTTTCTGGACATCT-3’  Reverse:5'-CACACGGTCTTCCACTTTGC-3’ |
| *Col1* | Forward:5'-ATGCCGCGACCTCAAGATG-3'  Reverse:5'-TGAGGCACAGACGGCTGAGTA-3' |
